# Curettage and bone grafting combined with electrocautery and drill, supplemented with plate fixation for the treatment of pediatric humeral aneurysmal bone cysts: a retrospective study

**DOI:** 10.3389/fped.2025.1673245

**Published:** 2025-10-15

**Authors:** Mengxue Liang, Jia Shi, Xuewen Ni, Li Zhang, Zuojia He, Jiaqi Chen, Hua Huang, Feifei Pu

**Affiliations:** ^1^ First Clinical College, Hubei University of Chinese Medicine, Wuhan, China; ^2^ Department of Orthopedics, Traditional Chinese and Western Medicine Hospital, Hubei University of Chinese Medicine, Wuhan, China; ^3^ Department of Orthopedics, Wuhan No.1 Hospital, Wuhan, China; ^4^ Department of Plastic Surgery, Central Hospital of Wuhan, Tongji Medical College, Huazhong University of Science and Technology, Wuhan, China; ^5^ Department of Orthopedics, Traditional Chinese and Western Medicine Hospital of Wuhan, Tongji Medical College, Huazhong University of Science and Technology, Wuhan, China

**Keywords:** pediatric, humerus, aneurysmal bone cyst, curettage and bone grafting, electrocautery, plate fixation

## Abstract

**Background:**

Aneurysmal bone cysts (ABCs) are benign, locally aggressive bone lesions that predominantly affect children and adolescents. The humerus is a common site, and treatment aims to eradicate the lesion while preserving growth potential and function. This study aimed to evaluate the clinical efficacy of curettage and bone grafting combined with electrocautery and burr drilling, supplemented with plate fixation, for treating pediatric humeral ABC.

**Methods:**

A retrospective analysis was conducted on 23 pediatric patients diagnosed with humeral ABC who underwent this surgical procedure. Inclusion criteria were age ≤18 years, a confirmed diagnosis via imaging and histopathology, and the absence of epiphyseal or joint surface involvement. The surgical techniques involved thorough curettage, electrocautery, burr drilling, bone grafting, and plate fixation. Postoperative follow-up included pain assessment using the Visual Analog Scale (VAS), functional evaluation using the Constant-Murley score, and radiographic imaging to assess bone healing and recurrence.

**Results:**

The average patient age was 8.6 years, and the mean follow-up period was 35.8 months. Postoperative pain scores (VAS) significantly decreased from a preoperative average of 5.6–1.1 at 1-year post-surgery (*P* < 0.05). Functional recovery, measured by the Constant-Murley score, improved from a preoperative average of 42–87 at 1-year post-surgery (*P* < 0.05). Radiographic evaluation confirmed complete lesion clearance and bone fusion in all patients, with no recurrence observed during the follow-up period. The overall complication rate was 17.39%, including one case of infection, two cases of wound dehiscence, and one case of transient radial nerve palsy, all of which were resolved with appropriate treatment.

**Conclusion:**

The combination of curettage, bone grafting, electrocautery, burr drilling, and plate fixation is a safe and effective treatment for pediatric humeral ABC. This approach ensures thorough lesion removal, promotes bone healing, and minimizes recurrence, making it a viable option for clinical application.

## Introduction

An aneurysmal bone cyst (ABC) is a relatively rare benign bone tumor-like lesion that accounts for approximately 1%–2% of primary bone tumors (1). It predominantly affects children and adolescents, with a predilection for the metaphyses of long bones, particularly the humerus (2). The exact etiology of ABC remains unclear; however, it is thought to be associated with local hemodynamic abnormalities, trauma, genetic factors, or secondary bone lesions (3). Pathologically, ABCs are characterized by blood-filled cystic cavities surrounded by fibrous tissue and multinucleated giant cells (4). Clinically, patients often present with localized pain, swelling, and limited mobility. In severe cases, pathological fractures may occur, significantly affecting limb function and quality of life (5).

The treatment of pediatric humeral ABC poses significant challenges in orthopedic practice. Given the ongoing skeletal growth in children, surgical intervention must balance between complete lesion removal and growth plate preservation to prevent impaired limb development (6, 7). Traditional treatment methods such as simple curettage, lesion resection, and radiotherapy have limitations. Simple curettage is associated with a 20%–30% recurrence rate, as reported in the literature (8). Although lesion resection can reduce recurrence, it may result in substantial bone defects, compromising skeletal stability (9). Radiotherapy, while historically used, is now generally avoided in the pediatric population due to the risks of growth plate damage and potential secondary malignant transformation (10). Therefore, the primary goals of treating pediatric humeral ABC are to reduce recurrence, promote bone healing, protect growth plates, and restore limb function effectively.

In recent years, advancements in surgical techniques and internal fixation materials led to the emergence of the combination of curettage, bone grafting, electrocautery and drill, and plate fixation as a promising approach for treating pediatric humeral ABC. The use of an electrocautery and drill allows for more thorough lesion removal, reduces residual tumor tissue, and lowers recurrence rates (11). Bone grafting fills defects and promotes bone healing, whereas plate fixation provides mechanical stability, prevents pathological fractures, and enables early functional rehabilitation (12). However, the long-term efficacy, complication rates, and impact on skeletal growth and development in children require further investigation.

This study aimed to retrospectively analyze cases of pediatric humeral ABC treated with curettage, bone grafting, electrocautery and drill, and plate fixation and to evaluate the clinical efficacy and safety of this approach.

## Methods

### Patients

A retrospective review was conducted on patients who underwent surgery for humeral ABCs between January 2015 and December 2022 at the Department of Orthopedics, Wuhan No.1 Hospital. This study was conducted in accordance with the Declaration of Helsinki and approved by the Ethics Committee of Wuhan No.1 Hospital (Approval No: TE-2024-50). The inclusion criteria were as follows: (1) Age ≤18 years, regardless of sex. (2) The diagnosis of humeral ABC was confirmed by imaging [radiography, computed tomography [CT], and magnetic resonance imaging [MRI]] and pathological examination. (3) Lesion located in the proximal humerus, shaft, or distal humerus without radiographic evidence of growth plate (physis) or articular surface involvement. (4) Treatment with curettage, bone grafting, electrocautery and drill, and plate fixation. (5) Informed consent obtained from the patient's guardians.

The exclusion criteria were designed to create a homogeneous cohort of primary, untreated ABCs without confounding pathologies, allowing for a clearer analysis of the outcomes of the described surgical technique. The exclusion criteria were as follows: (1) presence of other bone tumors (e.g., giant cell tumor, osteosarcoma) or systemic diseases (e.g., metabolic bone disease, infectious diseases). (2) Previous surgical treatment with recurrent or residual lesions. (3) Incomplete follow-up data or loss to follow-up.

### Surgical technique

Preoperative evaluations included blood tests, coagulation function tests, liver and kidney function tests, and electrocardiography. Imaging studies were used to assess the extent of the lesion, the degree of bone destruction, and its relationship with the surrounding tissues. A detailed surgical plan and contingency measures were discussed preoperatively.

Under general anesthesia, the patient was placed in a supine position with the affected limb abducted on a side table. The surgical principle of thorough curettage, electrocautery, drilling, grafting, and fixation was consistent for all patients. The specific type of bone graft (autograft, allograft, or synthetic) and the model of the plate (e.g., locking compression plate) were selected based on lesion size, bone stock, and surgeon preference. The surgical approach was selected based on the location of the lesion: the deltopectoral approach for proximal humeral lesions, the anterolateral approach for humeral shaft lesions, and the posterior approach for distal humeral lesions. After incising the skin and subcutaneous tissue, the muscles were dissected to expose the lesion. The lesion was thoroughly curetted while preserving the surrounding normal bone and growth plate. The cavity walls were cauterized using an electrocautery to eliminate residual tumor cells, and a drill was used to remove any remaining lesion tissues. Autologous iliac bone or allograft was trimmed and tightly packed into the cavity. To ensure stable fixation, a suitable plate (e.g., a locking plate or reconstruction plate) was selected based on the location of the lesion and bone quality. Hemostasis was achieved, and the wound was irrigated and closed in layers.

### Postoperative management

Postoperative monitoring included vital signs, limb circulation, sensation, and motor function. The incision was kept clean and dry, with regular dressing changes to prevent infection. Prophylactic antibiotics were administered for 3–5 days, and analgesics were given for pain relief. Early isometric exercises were encouraged to promote circulation and prevent muscle atrophy. Passive shoulder mobilization began at 4–6 weeks and progressed to active exercises. Strength training was initiated at 3 months. Follow-up imaging (radiography) was performed at 1, 3, 6, and 12 months to assess bone healing, plate position, and stability. CT or MRI was performed if needed to evaluate recurrence.

### Outcome measures

#### Surgical parameters

Operative time, intraoperative blood loss, hospital stay, and complications (e.g., infection, nerve injury, nonunion, and implant failure) were recorded. Intraoperative blood loss was calculated by measuring the volume of suction aspirate and subtracting the volume of irrigation fluid used, combined with the weight of saturated surgical sponges.

#### Pain assessment

Visual Analog Scale (VAS) scores were recorded preoperatively and at 1 week, 1 month, 3 months, 6 months, and 1 year postoperatively. The VAS, ranging from 0 (no pain) to 10 (worst pain imaginable), is a well-validated and routinely used tool for pain assessment in orthopedic department. It was administered by a nurse or therapist not involved in the surgery.

#### Functional recovery

The Constant-Murley score is a 100-point functional assessment tool for the shoulder, comprising domains for pain (15 points), activities of daily living (20 points), range of motion (40 points), and strength (25 points). A higher score indicates better shoulder function. Shoulder function was assessed using the Constant-Murley score preoperatively and at 1, 3, 6, and 12 months postoperatively.

#### Imaging evaluation

Radiography, CT, or MRI was used to assess the lesion clearance, bone graft fusion, plate position, and recurrence.

### Statistical analysis

Data were analyzed using SPSS software. Pairwise comparisons between preoperative scores and scores at each postoperative time point (1 week, 1, 3, 6, and 12 months) were performed using paired t-tests with Bonferroni correction for multiple comparisons. Statistical significance was defined as *p* < 0.05.

## Results

### Baseline characteristics

The study included 23 pediatric patients with humeral ABC, comprising 13 male and 10 female patients, aged 6–14 years (mean age: 10.32 ± 2.33 years). Lesions were located in the left humerus (11 patients) and right humerus (12 patients), with sizes ranging from 6.5 cm to 10.2 cm (mean: 8.4 ± 1.28 cm). The most common presenting symptom was pain (100%), followed by swelling (78%). Pathological fractures were present in 9 patients (39.1%). Preoperative imaging (radiography, CT, or MRI) revealed typical ABC features, such as expansile bone destruction and fluid-fluid levels. All lesions exhibited classic imaging features such as expansile, lytic lesions with thin sclerotic margins and fluid-fluid levels on MRI.

### Surgical outcomes

All surgeries were successfully completed, with operative times ranging from 65 to 140 min (mean: 95 ± 6.72 min) and intraoperative blood loss ranging from 75 to 230 ml (mean: 110 ± 8.45 ml). The lesions were thoroughly curetted, and the cavity walls were cauterized and drilled. Bone grafts included autologous bone, allografts, synthetic bone, or a combination of these. Plate fixation was selected based on the patient's age and lesion location.

### Postoperative follow-Up

All patients were followed for a minimum of 12 months postoperatively. All patients were followed up for 10–72 months (mean: 35.8 ± 5.36 months). Follow-up compliance was ensured through scheduled clinical and radiographic appointments, and telephone contact was used for patients who had moved.

#### Pain scores

The preoperative VAS score was 5.6 ± 1.18. Postoperative VAS scores significantly decreased over time, with values of 3.2 ± 0.85 at 1 month, 2.8 ± 0.24 at 3 months, 2.4 ± 0.32 at 6 months, and 1.1 ± 0.13 at 12 months (Table 1). Pairwise comparisons with Bonferroni correction revealed a statistically significant reduction in pain at each postoperative time point compared to preoperative levels (all *p* < 0.001). By 12 months postoperatively, 21 patients (91.3%) were pain-free (VAS = 0), and 2 patients (8.7%) reported only mild, non-disabling pain (VAS ≤ 2).

**Table 1:** Preoperative and postoperative clinical outcomes (mean ± SD) and pairwise comparisons.

Outcome measure	Preoperative	1 month	3 months	6 months	12 months
VAS score	5.6 ± 1.18	3.2 ± 0.85*	2.8 ± 0.24*	2.4 ± 0.32*	1.1 ± 0.13*
Constant-murley score	42 ± 6.81	54 ± 9.74**	65 ± 8.91*	76 ± 9.22*	87 ± 10.36*

* Significantly different from preoperative value (*p* < 0.001).

** Significantly different from preoperative value (*p* = 0.003).

#### Functional recovery

The preoperative Constant-Murley score was 42 ± 6.81. Functional scores showed significant improvement postoperatively, reaching 54 ± 9.74 at 1 month, 65 ± 8.91 at 3 months, 76 ± 9.22 at 6 months, and 87 ± 10.36 at 12 months (Table 1). Comparisons between preoperative and each postoperative time point were all statistically significant (preoperative vs. 1 month: *p* = 0.003; preoperative vs. 3 months: *p* < 0.001; preoperative vs. 6 months: *p* < 0.001; preoperative vs. 12 months: *p* < 0.001). At the final follow-up, 22 patients (95.7%) had excellent or good shoulder function, while 1 patient (4.3%) had a mild functional limitation.

#### Imaging findings

Postoperative radiography revealed complete lesion removal, satisfactory bone graft filling, and stable plate fixation. Bone grafts consisted of autologous iliac bone (*n* = 15), allograft (*n* = 5), synthetic bone (*n* = 2), or a combination of allograft and synthetic bone (*n* = 1). Bone healing was observed in 15 patients at 3 months, 18 patients at 6 months, and all patients at 12 months, with no recurrence. The mean time to radiographic fusion was 5.2 months for autografts and 5.8 months for allografts/synthetic grafts, with no statistically significant difference (*p* > 0.05).

#### Complications

**Figure 1:**
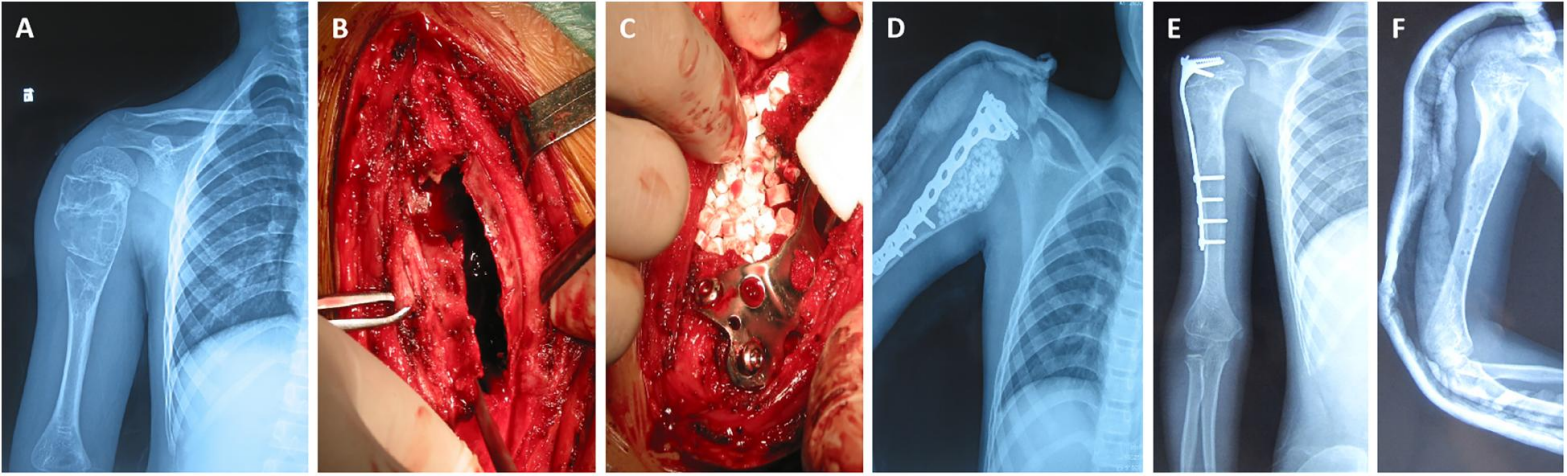
Curettage and bone grafting combined with electrocautery and drill, supplemented with plate fixation for the treatment of pediatric humeral aneurysmal bone cysts. **(A)** preoperative x-ray showed eccentric osteolytic changes; **(B)** curettage by electrocautery and drill, and **(C)** bone grafting; **(D,E)** x-ray at 1 week and 1 year postoperatively showed no complications such as hardware failure or bone resorption; **(F)** the plate fixation was removed at 2 years postoperatively.

The overall complication rate was 17.39%, including one case of incision infection, two cases of wound dehiscence, and one case of transient radial nerve palsy. The four complications were managed as follows: the superficial infection was resolved with a course of oral antibiotics; the two cases of minor wound dehiscence healed with local wound care and did not require surgical intervention; the case of transient radial nerve palsy recovered completely with neuropathic medication and physical therapy within 3 months. No cases of implant loosening or breakage were observed. No cases of postoperative frozen shoulder or significant joint stiffness were observed during the follow-up period. The rehabilitation protocol was deemed safe and effective. Figure 1 presents a typical case.

## Discussion

This study demonstrated that curettage, bone grafting, electrocautery and drill, and plate fixation are effective in treating pediatric humeral ABC, with significant pain relief, functional recovery, and low recurrence rates (13). Key surgical considerations include growth plate preservation, controlled use of an electrocautery and drill, and appropriate plate selection. Early postoperative rehabilitation is essential for optimal functional recovery.

The results of this study demonstrate that curettage and bone grafting combined with electrocautery burring and plate fixation showed significant clinical efficacy in treating pediatric humeral aneurysmal bone cysts. The adjunctive use of electrocautery and mechanical drilling may contribute to a reduced recurrence rate by aiding in the thorough eradication of the cyst lining, a outcome that can be challenging with curettage alone. The high-temperature effect of electrocautery burring not only inactivates residual tumor cells but also reduces intraoperative bleeding, improving the clarity of the surgical field (14). Additionally, adequate filling of bone graft materials provides a favorable biological environment for bone defect areas, promoting bone healing (15).

The use of plate fixation significantly enhances the mechanical stability of the humerus, particularly in highly active children, and effectively reduces the risk of postoperative fractures or implant failure (13). In this study, plate fixation was routinely employed for several reasons. First, it provides immediate mechanical stability, allowing for a more aggressive and thorough curettage without the concern of iatrogenic fracture. This is particularly important in the humerus, which is prone to torsion and bending forces. Second, it protects the bone graft, prevents collapse, and facilitates early postoperative mobilization, which is key to functional recovery in children. While implant removal may be required in the future, we believe the benefits of stability and enabling complete lesion eradication outweigh this potential secondary procedure. This approach is supported by recent literature advocating for stabilization in aggressive benign bone lesions to enable complete excision and early rehabilitation (16). Compared to simple curettage with bone grafting or external fixation, this combined surgical method demonstrates clear advantages in terms of functional recovery and radiographic outcomes.

Postoperative pain scores in children significantly decreased, and functional recovery was excellent, which is closely related to thorough lesion removal and stable internal fixation. Pain relief not only improved the quality of life of the children but also facilitated early functional rehabilitation. Regular postoperative functional exercises significantly improved shoulder joint range of motion and muscle strength. The Constant-Murley score, which comprehensively assesses shoulder function, demonstrated that the shoulder function of the children approached normal levels within one year of surgery. Furthermore, postoperative imaging revealed good bone graft fusion and stable plate positioning, with no significant stress shielding or loosening. These results further confirmed the superiority of this surgical method in promoting functional recovery.

Although this surgical method offers significant advantages, preventing complications remains a key clinical concern. This study did not report severe nerve injuries or infections, which may be attributed to meticulous intraoperative techniques and standardized postoperative management. While the use of electrocautery burring reduces the risk of bleeding, its high temperature may cause thermal damage to the surrounding tissues; therefore, its duration and extent of use must be carefully controlled during surgery. Although plate fixation provides mechanical stability, it carries the risk of stress concentration and stress shielding, particularly in children with incomplete skeletal development. Thus, close postoperative follow-up and regular imaging are necessary to promptly identify and address potential complications. In younger children, attention should be paid to the impact of the plate on epiphyseal growth, and absorbable materials or timely removal of internal fixation devices may be considered when necessary.

The use of an electrocautery and drill ensures thorough lesion removal and reduces the recurrence rate. Studies have reported recurrence rates of 10%–30% with traditional curettage, which decrease to <5% with the addition of an electrocautery and drill (17, 18). Bone graft selection is critical for healing. In this study, a 95% fusion rate was achieved using autologous bone, allografts, or synthetic bone. Plate fixation enhances mechanical stability, reduces fracture risk, and enables early rehabilitation.

This study has several limitations. Firstly, its retrospective design and lack of a control group (e.g., patients treated with curettage and grafting without fixation) limit the strength of our conclusions. Secondly, the sample size, though substantial for a rare condition, remains small. Thirdly, while our follow-up was sufficient to assess recurrence and healing, longer-term data are needed to fully evaluate the impact of the plate on the growing skeleton and the outcomes of eventual implant removal. Future prospective, comparative studies with longer follow-up are warranted.

## Conclusion

Curettage, bone grafting, electrocautery and drill, and plate fixation are safe and effective for treating pediatric humeral ABC, offering thorough lesion removal, high fusion rates, and low recurrence rates. Therefore, this method is recommended for clinical application.

## Data Availability

The original contributions presented in the study are included in the article/Supplementary Material, further inquiries can be directed to the corresponding author.
